# Selecting XFEL single-particle snapshots by geometric machine learning

**DOI:** 10.1063/4.0000060

**Published:** 2021-02-18

**Authors:** Eduardo R. Cruz-Chú, Ahmad Hosseinizadeh, Ghoncheh Mashayekhi, Russell Fung, Abbas Ourmazd, Peter Schwander

**Affiliations:** Department of Physics, University of Wisconsin-Milwaukee, 3135 N. Maryland Ave, Milwaukee, Wisconsin 53211, USA

## Abstract

A promising new route for structural biology is single-particle imaging with an X-ray Free-Electron Laser (XFEL). This method has the advantage that the samples do not require crystallization and can be examined at room temperature. However, high-resolution structures can only be obtained from a sufficiently large number of diffraction patterns of individual molecules, so-called single particles. Here, we present a method that allows for efficient identification of single particles in very large XFEL datasets, operates at low signal levels, and is tolerant to background. This method uses supervised Geometric Machine Learning (GML) to extract low-dimensional feature vectors from a training dataset, fuse test datasets into the feature space of training datasets, and separate the data into binary distributions of “single particles” and “non-single particles.” As a proof of principle, we tested simulated and experimental datasets of the Coliphage PR772 virus. We created a training dataset and classified three types of test datasets: First, a noise-free simulated test dataset, which gave near perfect separation. Second, simulated test datasets that were modified to reflect different levels of photon counts and background noise. These modified datasets were used to quantify the predictive limits of our approach. Third, an experimental dataset collected at the Stanford Linear Accelerator Center. The single-particle identification for this experimental dataset was compared with previously published results and it was found that GML covers a wide photon-count range, outperforming other single-particle identification methods. Moreover, a major advantage of GML is its ability to retrieve single particles in the presence of structural variability.

## INTRODUCTION

X-ray free-electron lasers (XFELs) generate femtosecond x-ray pulses with unprecedented intense brightness and high repetition rates, which have been used to determine biomolecular structures at high resolution and on ultra-short timescales.^1^ This remarkable advance recently opened a path for breakthrough research in structural biology.^2^ For example, serial-femtosecond crystallography (SFX) has been successfully employed to determine structures with near-atomic resolution and femtosecond time dynamics.^3,4^ However, SFX requires crystallization of the target biomolecules. An alternative XFEL technique is Single-Particle Imaging (SPI), where the samples are nebulized in tiny water droplets that progressively evaporate, exposing bare biomolecules to the x-ray pulse.^5^ Since SPI does not require crystallization, the biomolecules are not constrained in a packed lattice and can adopt different conformations. Moreover, as the samples are delivered at room temperature, SPI can also reveal molecular motions and movies^6^ that are not accessible by other structural methods that require low temperature, such as cryogenic Electron Microscopy (cryo-EM).

SPI with XFELs is still relatively new and under development,^7,8^ in particular, to improve its resolution to the subnanometer level. There are continuing upgrades in the instrumentation, such as high-throughput detectors to match the high-repetition rate of new generation of XFELs^9,10^ and state-of-the-art injector systems to deliver the biomolecules into the submicrometer-sized interaction region of the x-rays at high yield.^11,12^ Currently, at best, only about 2% of the XFEL pulses “hit” the sample^13–16^ and the injector developments primarily aim at increasing the hit rate. In parallel, data-analytic tools are being developed to efficiently identify, classify, and organize hundreds of thousands of diffraction patterns,^17–19^ preferably online during the beamtime in order to optimize the experimental parameters on the fly. Multiple successive steps constitute the workflow of the data processing pipeline: (1) hit finding, (2) single-particle identification, (3) particle orientation determination, (4) diffraction volume reconstruction, (5) conformational sorting, and (6) phase retrieval.^20–24^ Specifically, single-particle identification is critical for SPI, as high-resolution structure recovery requires a sufficiently large number of single-particle diffraction patterns.^25^ Nevertheless, XFEL datasets contain diffraction patterns of not only single particles but also multiple particles and other undesired objects and artifacts (water droplets, sample contaminations, erratic detector events, etc.). An ideal single-particle identification algorithm should reliably identify the subtle differences of these two groups and retrieve the desired single-particle diffraction patterns at low signal levels and high background noise. In this regard, initial effort was based on unsupervised classification methods, such as manifold embedding,^16,26^ which clusters similar diffraction patterns into regions in a low-dimensional feature space, and Principal Component Analysis (PCA) that quantifies correlations among diffraction patterns.^27,28^ It is worth mentioning a recent unsupervised method^29^ that employs an iterative expectation-maximization algorithm,^30^ similar to the ones used in single-particle cryo-EM to classify images.^31^ In fact, the data processing workflows for cryo-EM and SPI are quite similar, and both share a common requirement to achieve a high-resolution reconstruction: the single-particle identification step must retrieve a large number of snapshots, in order to overcome the noise from the low signal levels. More recently, single-particle identification methods are being implemented using supervised algorithms that require a known structural model^32^ or employ convolutional neural networks,^15,33,34^ which are based on training datasets with “ground-truth” information.

Here, we present a supervised single-particle identification method for XFEL datasets based on Geometric Machine Learning (GML). Our method efficiently identifies large numbers of single-particle diffraction patterns, outperforms previous methods at low signal levels, and is tolerant to background noise and intensity variations that arise from the sample-beam interaction point. Thus, it fulfills the most challenging requirements to reach high-resolution structural determination with SPI at XFELs. Our approach employs the Diffusion Map framework^35^ combined with Nyström out-of-sample extension^36^ to (1) define a low dimensional feature space from diffraction patterns in a training dataset, (2) fuse test datasets into the feature space of training datasets, and (3) convert the datasets into binary distributions of single particle and non-single particle diffraction patterns. The same methodology has already been successfully applied to a variety of problems that require accurate prediction from heterogeneous and incomplete data, such as lip reading^36^ and, most recently, fetal gestational age estimation.^37^

As a proof of principle, we tested the GML approach with simulated and experimental diffraction patterns of the Coliphage PR772 virus, a 70-nm-diameter biomolecular assembly. We report two different metrics to evaluate the quality of the GML predictions: the “recall,” which is the ratio between selected single particles and the total number of single particles in the dataset, and the “precision,” which is the ratio of the correctly predicted single particles and the total number of selected snapshots. All our predictions employed a noise-free simulated dataset for training. First, we evaluated noise-free test datasets of simulated diffraction patterns of PR772 consisting of single and multiple particles and obtained a single-particle identification with a precision of 95% and a recall of 99%. Second, we modified the simulated test datasets by varying the photon count, noise levels, background signal, and intensity from the sample-beam interaction point. All these factors decrease both the precision and the recall down to 82%. Finally, we applied the method to an experimental XFEL dataset showing a single-particle precision of 85% and a recall of 56% and discuss putative improvements. Overall, our results demonstrate that GML is a promising and efficient data analysis technique for single-particle identification of large datasets.

## METHODS

### Geometric machine learning

The Geometric Machine Learning (GML) approach is based on manifold-based embedding, a method that has been successfully applied for orientation recovery^38,39^ and for sorting different types of molecular conformations.^40,41^ Here, we focused on a new application, that is, single-particle identification. As previously reported using simulated and experimental data,^6,42^ manifold embedding represents high-dimensional diffraction patterns in terms of low-dimensional eigenvectors of a similarity matrix, so-called diffusion map. Thus, diffraction patterns with similar features also have similar eigenvector projections and are located close to each other in the manifold space. Then, an expert visually inspects the different regions of the manifold space to identify which region contains primarily single-particle hits. A drawback of this approach is that any refinement to narrow the single-particle region requires re-computing the diffusion map, which also needs a new parameter optimization and subsequent visual identification by an expert. We rectified this approach into an automated sorting procedure that transforms the manifold representation into a one-dimensional bimodal distribution of single particles and non-single particles. For this purpose, we incorporated a Nyström extension approach^36^ into the diffusion map framework.^35^ Our protocol employs the information of the embedding manifold Ψ of a training dataset and a binary ground truth vector f to compute a transform vector C. The vector C is subsequently used to obtain a prediction vector $\tilde{f}$ for the extended manifold of a test dataset. The protocol is described as follows.

First, a training dataset of diffraction patterns is embedded by the diffusion map into a low-dimensional space,

$$\Psi =\left[\begin{array}{ccc} \psi_{1,1} & \cdots & \psi_{1,k}\\ \vdots & \ddots & \vdots \\ \psi_{n,1} & \cdots & \psi_{n,k} \end{array}\right].$$ (1)

Here, n is the number of samples and k is the dimension of the embedding space (k≪n). That is, the manifold is described by the matrix Ψ, and each row represents a diffraction pattern in the embedding space spanned by k eigenvectors. Additionally, a vector f is defined based on the ground truth information of the training dataset. The components of f are provided by the user; we chose a binary classification that takes a value “1” for single particles and “0” for non-single particles.

Second, we define a vector C, which yields f when transformed by matrix Ψ,

$$\left[\begin{array}{ccc} \psi_{1,1} & \cdots & \psi_{1,k}\\ \vdots & \ddots & \vdots \\ \psi_{n,1} & \cdots & \psi_{n,k} \end{array}\right]\left[\begin{array}{c} C_{1}\\ \vdots \\ C_{k} \end{array}\right]=\left[\begin{array}{c} f_{1}\\ \vdots \\ f_{n} \end{array}\right].$$ (2)

The vector C is determined as follows:

$$\left[\begin{array}{c} C_{1}\\ \vdots \\ C_{k} \end{array}\right]={\left[\begin{array}{ccc} \psi_{1,1} & \cdots & \psi_{1,k}\\ \vdots & \ddots & \vdots \\ \psi_{n,1} & \cdots & \psi_{n,k} \end{array}\right]}^{T}\;\left[\begin{array}{c} f_{1}\\ \vdots \\ f_{n} \end{array}\right].$$ (3)

Here, we used the identity $\Psi^{T}\Psi =1$, a consequence of the eigenvectors being orthonormal.

Third, a test dataset containing m diffraction patterns is embedded into the k-dimensional extended manifold $\tilde{\Psi }$, with

$$\tilde{\Psi }=\left[\begin{array}{c} {\tilde{\Psi }}_{1}\\ \vdots \\ {\tilde{\Psi }}_{m} \end{array}\right]=\left[\begin{array}{ccc} {\tilde{\psi }}_{1,1} & \cdots & {\tilde{\psi }}_{1,k}\\ \vdots & \ddots & \vdots \\ {\tilde{\psi }}_{m,1} & \cdots & {\tilde{\psi }}_{m,k} \end{array}\right].$$ (4)

To proceed, we follow the Nyström extension as described by Lafon *et al.*^36^ The Euclidian distances between each test diffraction pattern and the training dataset are calculated. Those distances are then used to project each test diffraction pattern into the manifold space of the training set.

Finally, C is used to estimate the prediction vector $\tilde{f}$ for the test dataset,

$$\left[\begin{array}{ccc} {\tilde{\psi }}_{1,1} & \cdots & {\tilde{\psi }}_{1,k}\\ \vdots & \ddots & \vdots \\ {\tilde{\psi }}_{m,1} & \cdots & {\tilde{\psi }}_{m,k} \end{array}\right]\left[\begin{array}{c} C_{1}\\ \vdots \\ C_{k} \end{array}\right]=\left[\begin{array}{c} {\tilde{f}}_{1}\\ \vdots \\ {\tilde{f}}_{m} \end{array}\right].$$ (5)

Thus, $\tilde{f}$ contains the predictions for all diffraction patterns of the test dataset; single particles should yield predictions near 1 and non-single particles near 0. A schematic workflow of the GML method and the computational cost of each step are presented in the supplementary material—Sec. S7.

### Diffraction datasets

The GML approach was first evaluated using simulated XFEL datasets. After that, the GML approach was applied to an experimental XFEL dataset, which was collected at the Linac Coherent Light Source (LCLS) facility at the Stanford Linear Accelerator Center (SLAC).^16^

For the simulated datasets, the diffraction patterns for single and multiple particles were generated from a previously reported diffraction volume.^6^ The diffraction patterns were simulated using the same parameters as in the experiment, namely, a photon energy of 1.6 keV and the resolution at the detector corner was 9.0 nm (q = 0.11 nm^−1^). The distribution of single and multiple particles was obtained from Poisson statistics (see the supplementary material—Sec. S1). Two simulated datasets were created: “training” and “test.” The training dataset consists of 80 000 noise-free diffraction patterns (46 653 single particles and 33 347 multiple particles). The number of single particles was chosen to give a representative distribution of single-particle diffraction patterns of the structure (supplementary material—Sec. S2). The test dataset consisted of 100 000 diffraction patterns (58 488 single particles and 41 512 multiple particles). The total number of diffraction patterns was chosen to be similar to the dataset sizes in current experiments,^14,16^ that is, on the order of a hundred thousand. The diffraction patterns in the test dataset were modified using different levels of photon count and background, including intensity variations that arise at the interaction point, in order to mimic diffraction patterns corresponding to realistic experimental situations.

**FIG. 1.**
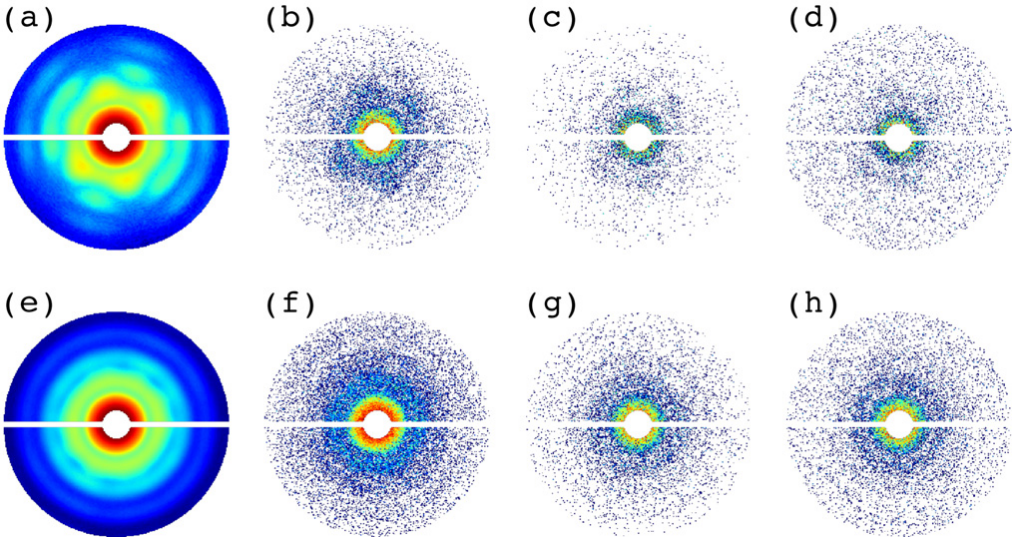
Test datasets with different modifications. The first row shows (a) a noise-free single-particle diffraction pattern and modified to obtain ${\left\langle n_{ph}\right\rangle }_{Sh}$ values of (b) 1 (c), 0.1, and (d) 0.1 with the background signal at b=10. The second row shows a noise-free multiple-particle diffraction pattern obtained by adding seven random single-particle diffraction patterns: (e) noise-free and modified to obtain ${\left\langle n_{ph}\right\rangle }_{Sh}$ values of (f) 1, (g) 0.1, and (h) 0.1 with the background signal at b=10. The horizontal gap in each diffraction pattern emulates the gap between two detector panels. To enhance visual contrast and highlight the diffraction features, photon counts shown have been scaled logarithmically, and inner and outer circular masks corresponding to q values <0.001 nm^−1^ and >0.081 nm^−1^ have been applied.

Figure 1 shows some typical snapshots from the test dataset after the application of the modifications. To vary the photon count scattered from the sample, a scaling factor α was applied to the entire diffraction pattern. α controls the average number of photons per Shannon pixel in the outer disk region ($q\in \left[0.06,\;0.08\right]$ nm^−1^). We abbreviated this average as ${\left\langle n_{ph}\right\rangle }_{Sh}$, and it was used to characterize photon counts for the different test datasets. We used ${\left\langle n_{ph}\right\rangle }_{Sh}$ values of 0.001, 0.01, 0.1, and 1. To simulate the background, we considered the x-ray scattering factor from a helium gas, which turns out to be almost constant in the q-range used. Thus, a constant background was added to the scattering signal of the sample. The background signal had a ${\left\langle n_{ph}\right\rangle }_{Sh}$ value similar to the sample, multiplied by a factor b. We chose b with the following values: 0 (no background, only sample signal), 1 (background signal = sample signal), 10, and 100. To account for variations due to the source and shot-to-shot impact conditions, an additional factor w was used to vary the ${\left\langle n_{ph}\right\rangle }_{Sh}$ value of each diffraction pattern, where w is a random number between 0.2 and 1.8. After applying all these factors, diffraction patterns were generated according to Poisson statistics.

For the experimental test dataset, we used a PR772 dataset that has been deposited into the Coherent X-ray Imaging Data Bank^43^ (CXIDB 58). This dataset consists of 135 375 identified hits collected at the Atomic Molecular Optics (AMO) beamline at LCLS.^16^ The diffraction snapshots were pre-processed as described by Hosseinizadeh *et al.*,^6^ which includes removing outlier patterns with high photon counts, resulting in a final test dataset of 133 900 diffraction patterns. The ${\left\langle n_{ph}\right\rangle }_{Sh}$ value for the experimental data is 0.78. For both experimental and simulated diffraction patterns, we considered an annular disk within a q-range of $\left[0.03,\;0.08\right]$ nm^−1^ for manifold embedding. Further details of the pre-processing steps for experimental and simulated datasets are provided in the supplementary material—Sec. S3.

### Metrics for prediction quality

The simulated datasets were employed to evaluate single-particle prediction by GML. To this end, the ground truth information of the test datasets and the prediction vector $\tilde{f}$ was used to plot Receiver Operating Characteristic (ROC) curves,^44^ which provide an estimate for the ratio of single and multiple particles correctly predicted. Alternatively, we also reported the extent to which a predicted subset contains single particles only, what we have termed “purity plots.” Each component of $\tilde{f}$ represents the prediction for a diffraction pattern. Thus, we used $\tilde{f}$ to define a cutoff criterion for optimal single-particle selection. First, the deviation (d) from the ideal prediction of a single-particle diffraction pattern is computed as

$$d=\left|\tilde{f}-1\right|.$$ (6)

A single-particle prediction ($\tilde{f}\approx 1$), thus, has the d value near zero; conversely, a multiple-particle prediction ($\tilde{f}\approx 0$) has a d value near 1. Second, the diffraction patterns were sorted according to ascending d values. Third, an integer index was assigned to each diffraction pattern based on the sorted d values. Finally, the first τ indices were designated as single-particle diffraction patterns. As the cutoff criterion τ was varied from 1 to m (the entire number of patterns in the dataset), more single-particle and multiple-particle diffraction patterns were included in the subset selection. We then evaluated the subsets using ROC and purity plots.

ROC curves show the false positive rate (FPR) vs the true positive rate (TPR),

$$\begin{array}{c} F\mathrm{PR}\left(\tau \right)=\frac{\mathrm{FP}(\tau )}{N},\\ T\mathrm{PR}\left(\tau \right)=\frac{\mathrm{TP}(\tau )}{P}, \end{array}$$ (7)

where TP(τ) and $FP\left(\tau \right)$ are the numbers of single particles (true positives) and multiple particles (false positives) for a given τ cutoff. P and N are the total number of single particles and multiple particles in the test datasets, respectively. The FPR appears as the abscissa axis; thus, the low content of false positives populates the left side of the ROC curve. The TPR appears as the ordinate axis, that is, the high content of true positives is located near the top. Thus, the more sharply the ROC curve bends toward the upper left corner, the better the method.

The true positive rate (TPR) is also known as recall,^44^ a metric we report to identify the percentage of single particles retrieved from the dataset. Another metric used in the GML diagnosis is the precision,^44^ which accounts for the ratio of correctly predicted single particles in a subset selection, and it is defined as follows:

$$\mathrm{precision}\left(\tau \right)=\frac{\mathrm{TP}\left(\tau \right)}{\mathrm{TP}\left(\tau \right)+\mathrm{FP}(\tau )}.$$ (8)

Finally, a purity plot is defined as

$$\chi \left(\tau \right)=\frac{\mathrm{TP}(\tau )}{P}\;\left[1-\frac{\mathrm{FP}(\tau )}{\mathrm{TP}(\tau )}\right].$$ (9)

The first term is the true positive rate (TPR). The second term is a “contamination factor” that considers the multiple particles within the selection. This equation can be rewritten as

$$\chi \left(\tau \right)=\frac{\mathrm{TP}\left(\tau \right)-\mathrm{FP}\left(\tau \right)}{P}.$$ (10)

Thus, an optimal subset selection should have χ≈1, which occurs when $TP\left(\tau \right)\approx P$ and $FP\left(\tau \right)\approx 0.$

## RESULTS

### Training dataset

In this section, we present the embedding of the training dataset and a validation of the GML method. First, we describe the parameter space that controls the shape of the diffusion map. Then, we used the training dataset composed of 80 000 diffraction patterns to compute a diffusion map and its corresponding transform column vector C for different parameter combinations. After that, we used the same training dataset to compute a prediction vector $\tilde{f}$ for each parameter combination. Finally, each prediction vector $\tilde{f}$ is compared with the ground truth vector f using ROC and purity plots.

**FIG. 2.**
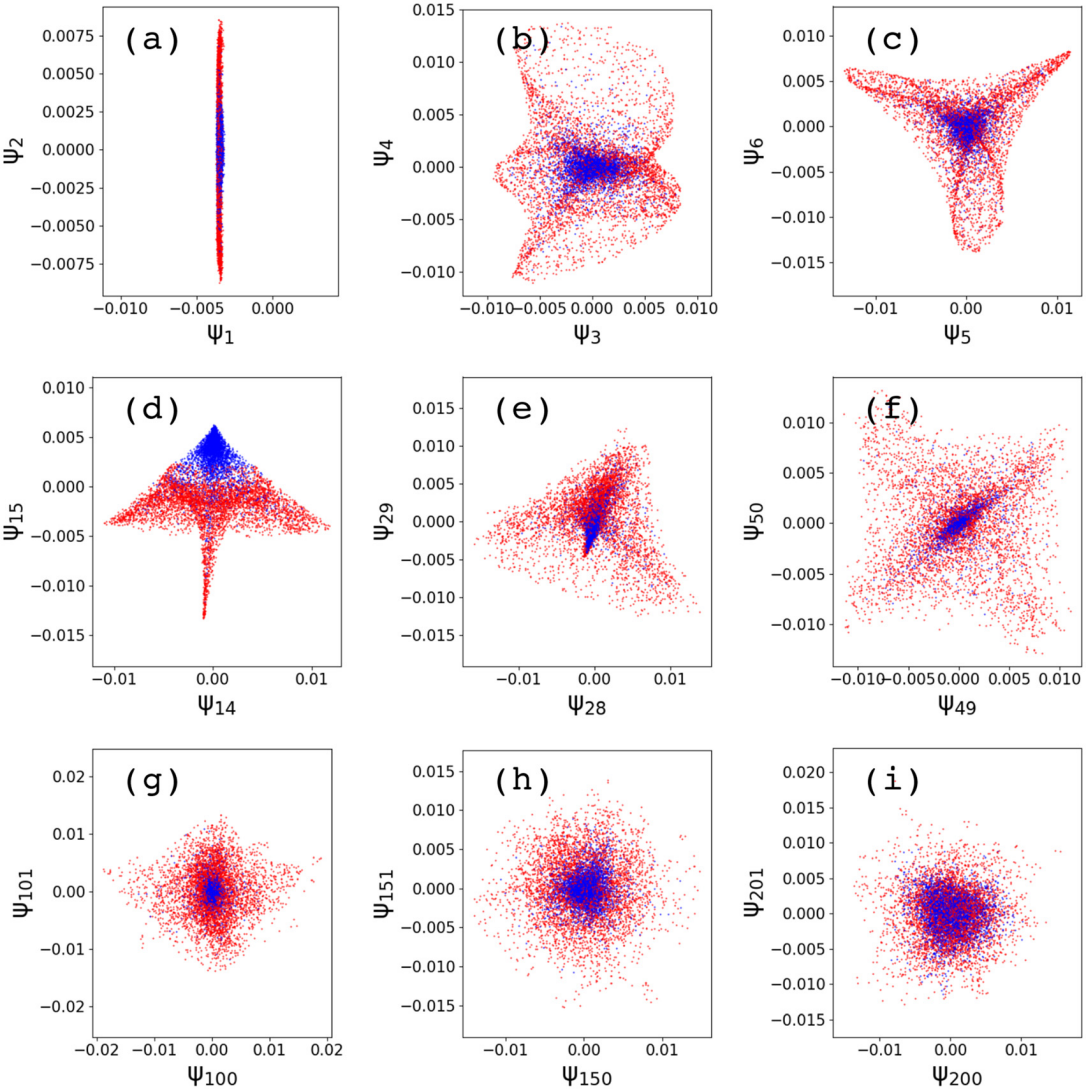
Diffusion map analysis of the training dataset. Each panel shows a different two-dimensional projection of the manifold of the noise-free dataset composed of 80 000 diffraction patterns. Diffusion map parameters used here are k=201, nN = 30, and $\sigma_{N}$ = 10 * $\sigma_{F}$. Single particles are colored in red and multiple particles in blue.

In manifold embedding calculations by the diffusion map, three parameters are used to compute the training manifold: the dimension of the embedding space k, the number of nearest-neighbors nN, and the width of the kernel $\sigma_{N}$.^35,36^ To test the robustness of our approach, our calculations have been repeated for a wide range of parameters. The number of eigenfunctions k ranges from 1 to 201. The number of nearest neighbors nN goes from 10 to 1000; for each nN value, we calculated the optimal length scale $\sigma_{F}$, as described by Ferguson *et al.*^45^ The kernel width $\sigma_{N}$ goes from 10*$\sigma_{F}$ to 1000*$\sigma_{F}$. We considered as optimal parameter combination the one that generates the sharpest bimodal distribution of the prediction vector $\tilde{f}$ (see below). Figure 2 shows various two-dimensional diffusion-map eigenvector projections of the embedded training dataset for a representative set of parameters. Even though single particles (red) and multiple particles (blue) appear throughout the manifold space, they preferentially populate different regions in the manifold.

**FIG. 3.**
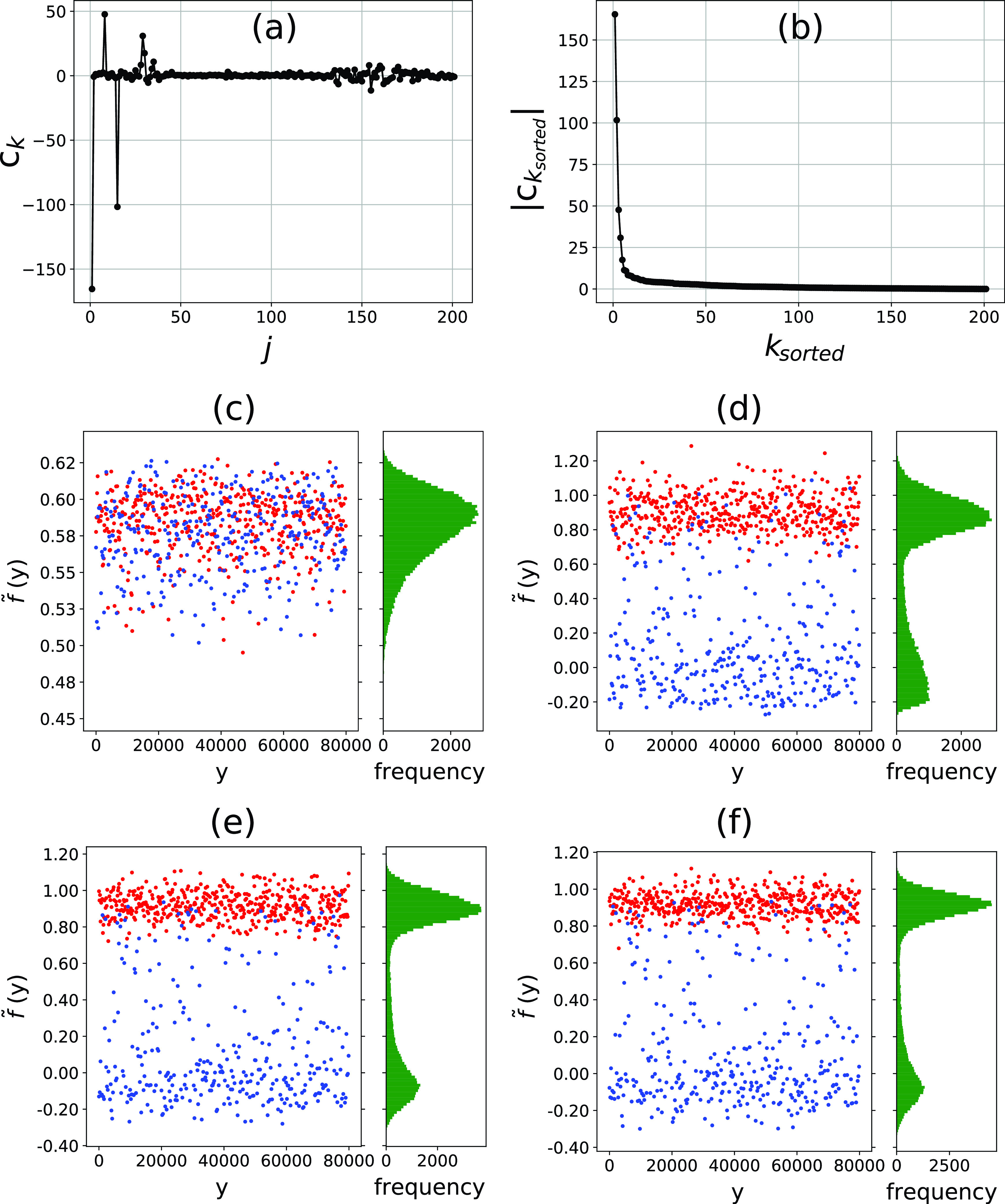
GML predictions for the training dataset. Panels (a) and (b) show the components $C_{k}$ [Eq. (3)] unsorted and sorted according to their absolute values, respectively. Panels (c)–(f) show the prediction values $\tilde{f}$ for single particles (red) and multiple particles (blue) using (c) $k_{\mathrm{sorted}}=1$, (d) $k_{\mathrm{sorted}}=10$, (e) $k_{\mathrm{sorted}}=50$, and (f) $k_{\mathrm{sorted}}=201$. Histograms show that an initially monomodal distribution separates into a bimodal distribution; which sharpens as the number of eigenvectors increases. See the accompanying animation in the supplementary material.

After embedding the training dataset, we used the resulting diffusion maps and ground truth information f to estimate the vector C [Eq. (3)]. Figure 3(a) shows that the $C_{k}$ values are not monotonic in k. To investigate which coefficients $C_{k}$ are contributing the most to the prediction, we sorted them based on their absolute values ($C_{k_{\mathrm{sorted}}}$). As Fig. 3(b) shows, the re-ordering from $C_{k}$ to $C_{k_{\mathrm{sorted}}}$ reveals that about a dozen $C_{k_{\mathrm{sorted}}}$ are the most significant.

To validate the predictive capabilities of the GML method, we computed the prediction vector $\tilde{f}$ for the training dataset itself. Figures 3(c)–3(f) illustrate how the magnitudes of the coefficients $C_{k_{\mathrm{sorted}}}$ affect the contributions of $\Psi_{k_{\mathrm{sorted}}}$ and, therefore, the prediction value $\tilde{f}$. When using only the first dominant eigenvector [Fig. 3(c)], there is no separation between single and multiple particles. As the number of eigenvectors increases to 10 [Fig. 3(d)], a bimodal distribution appears, with single particles around $\tilde{f}\approx 1$ and multiple particles around $\tilde{f}\approx 0$. Adding more eigenvectors up to 50 [Fig. 3(e)] and 201 [Fig. 3(f)] results in small improvements that sharpen the peaks. Thus, although the most significant contributions come from the first dozen $C_{k_{\mathrm{sorted}}}$, the other coefficients provide small improvements to the quality of separation. An animation showing the evolution of $\tilde{f}$ with k is given in the supplementary material—Sec. S4. Since there is no distortion of the bimodal distribution with the increasing eigenvector index, we used the entire range of 201 eigenvectors to compute $\tilde{f}$.

**FIG. 4.**
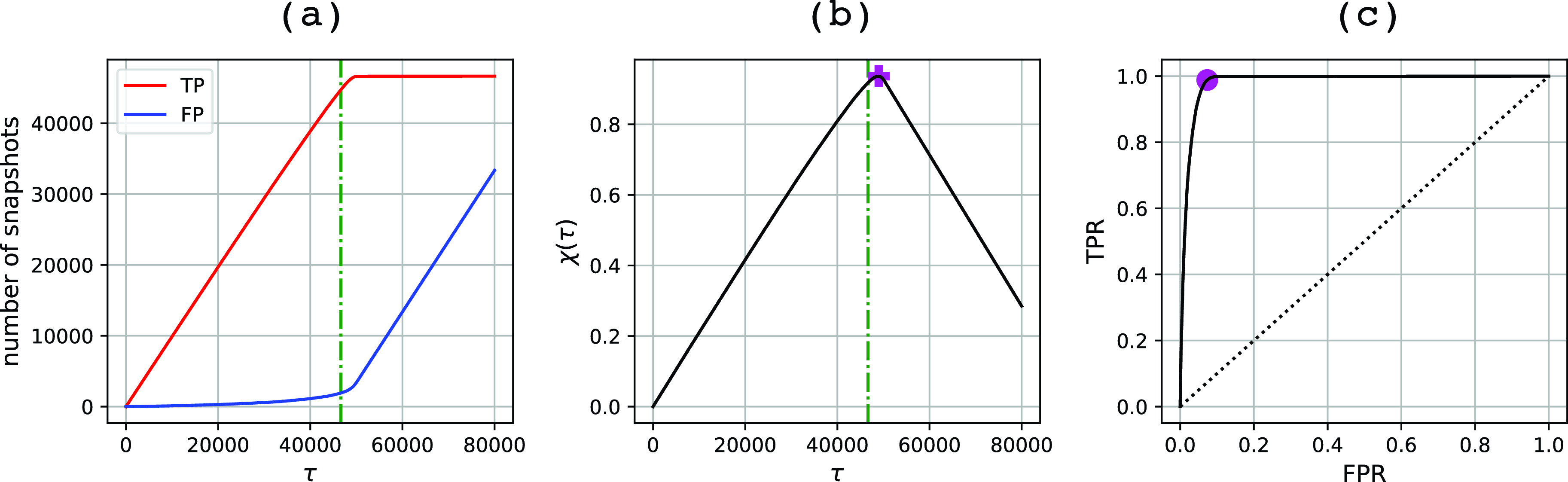
Purity and ROC curves for the training dataset. The plots refer to GML predictions using the training dataset as the test dataset. Panel (a) shows the variation of singles (TP) and multiple (FP) particles selected at cutoff value τ. The training dataset contains 46 653 single particles and 33 347 multiple particles. Panel (b) shows a purity plot, and the purple cross marks the highest point on the curve with τ = 49 004 and χ = 0.94. Vertical green lines in (a) and (b) at τ = 46 653 have been added as guides to the eye. Panel (c) shows the ROC curve, and the purple dot marks the furthest point to the diagonal with FPR = 0.07 and TPR = 0.99.

Finally, we analyzed the GML predictions using ROC and purity plots. Figure 4(a) shows the numbers of single and multiple particles included in the subset selection as the cutoff criterion τ varies. The green vertical line is a guideline located at τ=P. In a perfect identification scheme, the single-particle count (red line) would steadily increase for τ≤P and the multiple particles (blue line) would appear only after τ>P. Our results follow a similar scenario: the single particles appear at low τ values, and multiple particles enter at higher cutoffs when most of the singles have already been found. However, there is a certain degree of mixing that hinders a perfect separation. Accordingly, the purity plot and ROC curves show a good separation. The purity plot [Fig. 4(b)] shows a steady increase with τ, reaching its maximum value at τ=49 004 (46 347 single particles and 2657 multiple particles) with a precision of 94.58% and a recall of 99.34%. Obviously, there is an inevitable trade-off between recall and purity; at lower τ values, the subsets contain not only a higher precision but also a lower recall of single particles. The ROC curve shows similar results [Fig. 4(c)]. It displays a sharp increase near the left border, the region with very few false positives, reaching a point close to the top left corner (purple dot) that corresponds to a subset containing 48 560 diffraction patterns with a precision of 94.95% and a recall of 98.83%.

### Simulated test dataset

The GML method was evaluated further using test datasets that were modified with different photon counts and variations in the background signal. We characterize the quality of each GML prediction by the recall and the precision of the subset located at the furthest point to the diagonal line of the ROC curve, which represents the best compromise between precision and recall. We termed the subset selection at that point the “best prediction” for a given ROC curve.

**FIG. 5.**
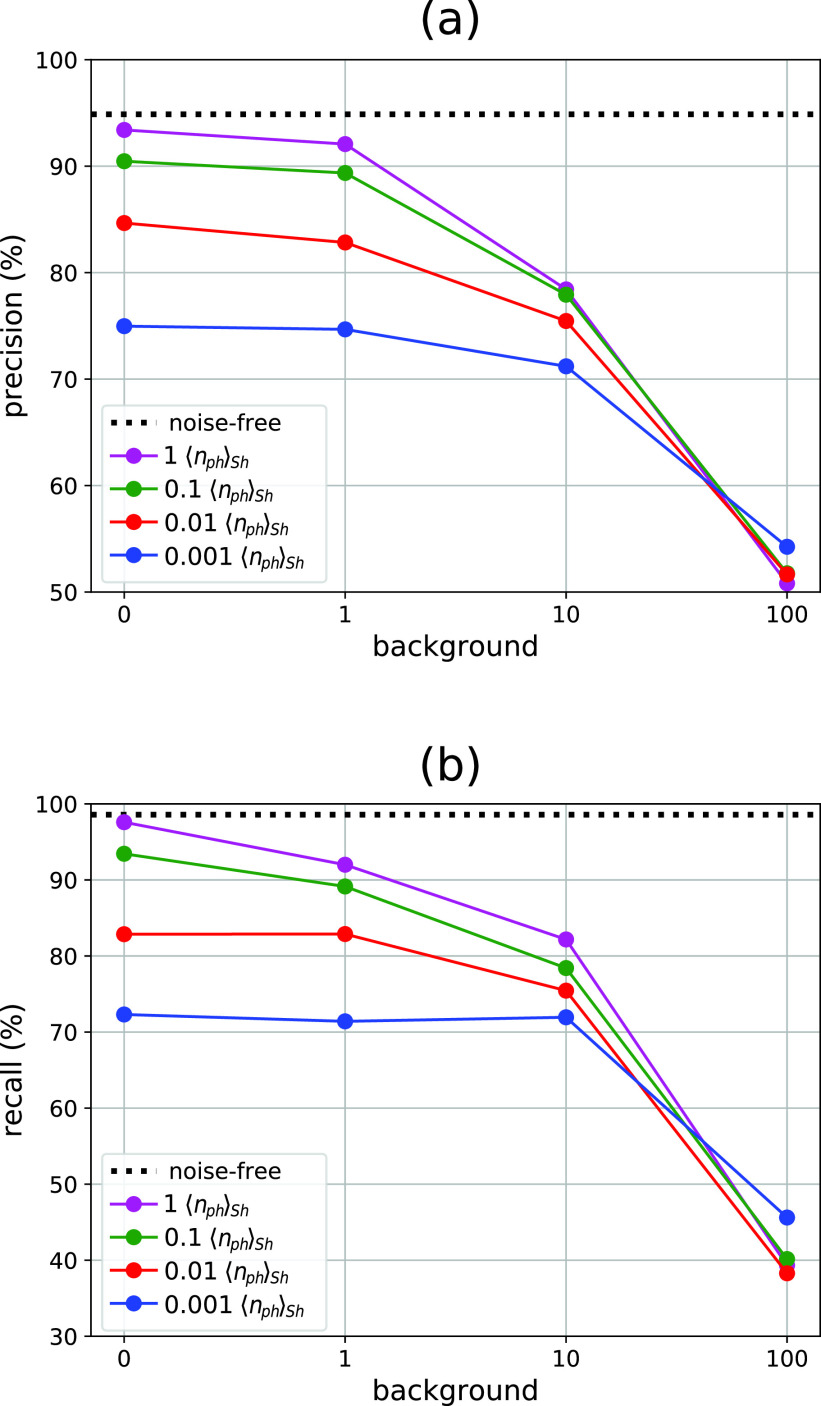
Quality of the GML predictions for noisy simulated test datasets. The panels quantify the best prediction subset based on ROC plots for different magnitudes of photon counts (color lines) and background noise (abscissa axis). Panel (a) shows the precision as percentage. Panel (b) shows the percentage of recall. Horizontal dotted lines refer to values for the noise-free test dataset. ${\left\langle n_{ph}\right\rangle }_{Sh}$ is an abbreviation for the average number of photons per Shannon pixel in the outer disk region ($q\in \left[0.06,\;0.08\right]$ nm^−1^).

A noise-free collection of 100 000 diffraction patterns was used here as the test dataset. This noise-free test dataset was evaluated against the training dataset of 80 000 diffraction patterns using the GML method, which outputs a best-prediction subset of 60 774 diffraction patterns with a precision of 94.95% and a recall of 98.58%. After that, the test dataset was modified by inclusion of Poisson noise to mimic four levels of average photon counts and four levels of the background signal (see the Methods). Figure 5 summarizes the results. Individual ROC and purity plots are provided in the supplementary material—Sec. S5. First, we discuss the diffraction patterns that have ${\left\langle n_{ph}\right\rangle }_{Sh}$ values from 0.01 to 1 (red, green, and purple colors). As expected, both increasing background noise and decreasing photon signals gradually reduce the best-prediction metrics. For background signals lower than or similar to the particle diffraction signal (b≤1), the best-prediction subsets result in values of 82.89% for recall and 82.83% for precision (abscissa axes in Fig. 5). For a background ten times higher than the particle signal (b=10), the subset's recall and precision go down to 75.44% and 75.46%, respectively. An extreme case with the background one hundred times higher (b=100) results in a subset with a recall of 38.28% and a precision of 51.66%, that is, the diffraction patterns are saturated by the background signal and the GML method cannot identify single particles. Thus, the predictive region for the simulated datasets covers background signals up to the order of the diffraction signal for the maximum resolution used (q<0.08). Within this region, the datasets with ${\left\langle n_{ph}\right\rangle }_{Sh}$ values of 1 and 0.1 result in subset selections with values of recall and precision near or above 90%. A low particle signal with an ${\left\langle n_{ph}\right\rangle }_{Sh}$ value of 0.01 decreases both the recall and the precision down to about 83%.

Finally, we tested an extremely low signal case, a dataset with an ${\left\langle n_{ph}\right\rangle }_{Sh}$ value of 0.001 (blue color in Fig. 5). For background intensities of b = 0, 1, and 10, the precision progressively decreases to 74.97%, 74.67%, and 71.20%, respectively, whereas the recall decreases to a similar value of about 72%. Thus, for the last example, the GML method can still output subsets with a higher proportion of singles than of multiple particles, but further refinement would be required to increase the precision. As before, for a background of b = 100, both the precision and the recall drop significantly to 54.24% and 45.61%, respectively, that is, the background signal degrades the GML predictions.

### Experimental dataset

In this section, we apply the GML method to make predictions for an experimental PR772 dataset.^43^ First, we compute the prediction vector $\tilde{f}$ and discuss its effectiveness to identify diffraction patterns of single particles in an experimental dataset. Second, we correlate the values of the prediction $\tilde{f}$ with the results from three other methods of single-particle identification, all of them applied on the same experimental dataset. Third, we use a small subset of diffraction patterns manually classified by an expert to compare the advantages and disadvantages of GML and other single-particle identification methods.

**FIG. 6.**
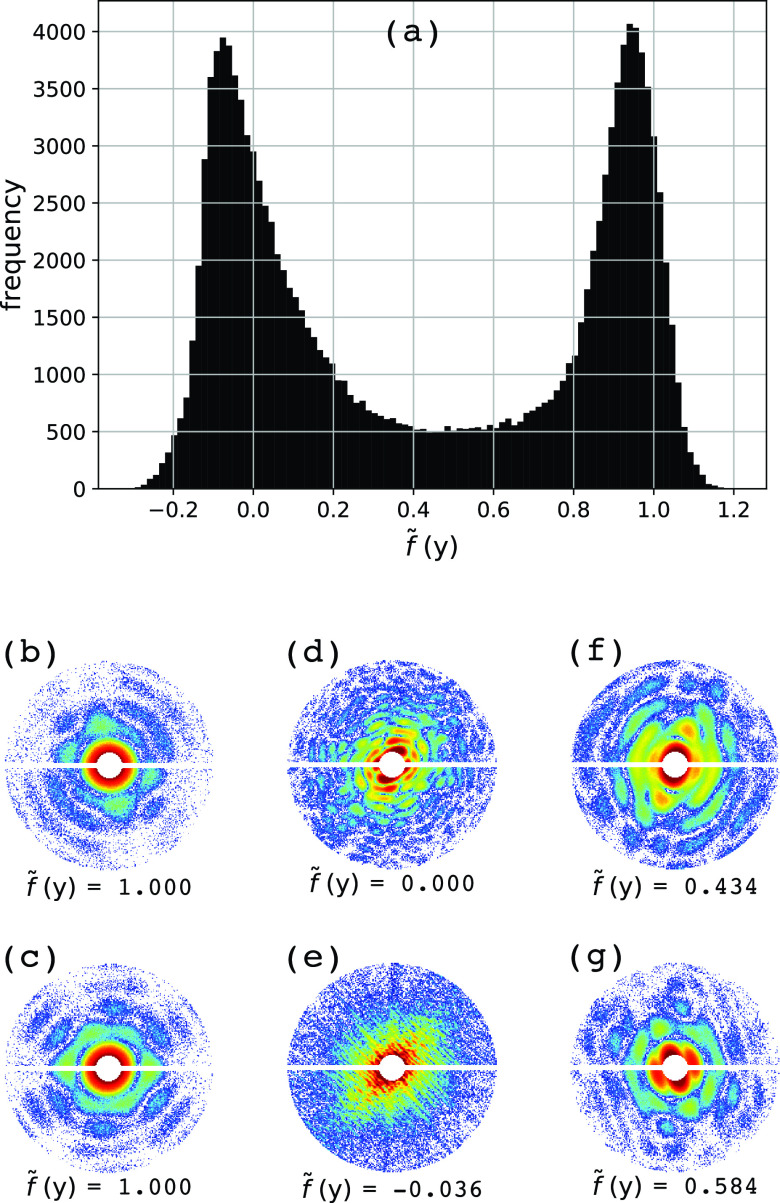
GML prediction for the experimental dataset. Panel (a) shows the histogram for the predicted $\tilde{f}$ values for an experimental dataset of 133 900 diffraction patterns. Snapshots (b)–(g) show six diffraction patterns and their corresponding $\tilde{f}$ values.

Figure 6(a) shows that the prediction $\tilde{f}$ for the experimental dataset has a bimodal distribution that is similar to the ones of the simulated datasets, with one peak near the region of single particles ($\tilde{f}\approx 1$) and the other peak near the region for multiple particles ($\tilde{f}\approx 0$). Figures 6(b)–6(g) show diffraction patterns located in different regions of the histogram. At first glance, the experimental diffraction patterns located near $\tilde{f}\approx 1$, [Figs. 6(b) and 6(c)] closely resemble the simulated diffraction patterns of single particles. Due to the relatively high photon counts of the experimental dataset (${\left\langle n_{ph}\right\rangle }_{Sh}=0.78$), it is expected that GML would result in a good prediction for single-particle identification. Conversely, the experimental diffraction patterns located near $\tilde{f}\approx 0$, [Figs. 6(d) and 6(e)] reveal diffraction subtleties that we did not consider in the simulated datasets, such as undulated patterns with fringes due to coherent interparticle interference [Fig. 6(d)]. Moreover, the experimental dataset also contains random scattering events [Fig. 6(e)], e.g., due to water droplets or erratic detector artifacts, but they are still correctly identified as non-single particles. Finally, for the diffraction patterns located in the middle region between the histogram peaks [Figs. 6(f) and 6(g)], we can recognize a mixture of features from single and multiple particles.

**FIG. 7.**
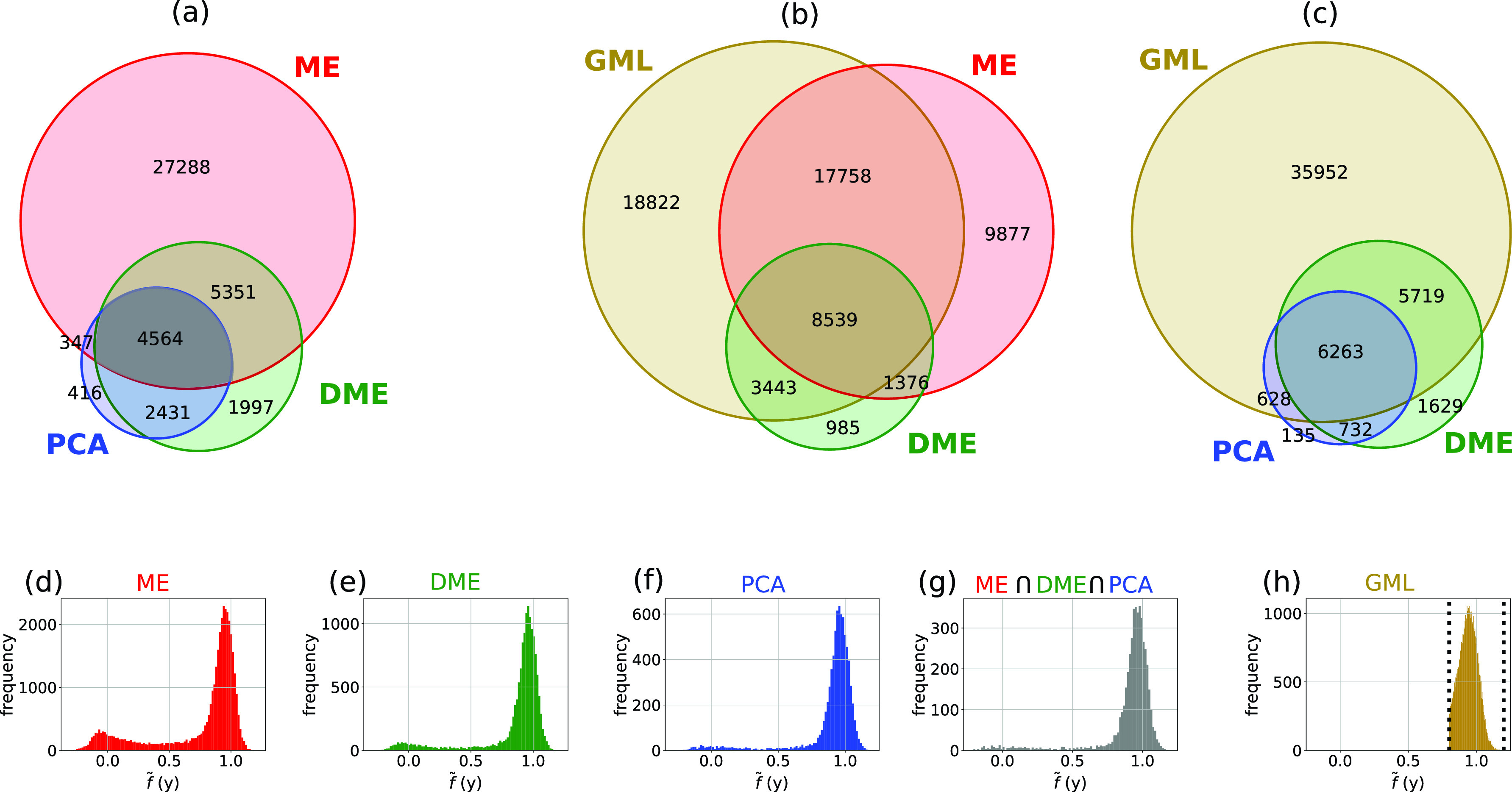
Different classifications for single-particle identification of the experimental dataset of PR772 virus. Panels (a)–(c) show Venn diagrams for four subsets of single-particle identification. Principal Component Analysis (PCA—blue), Diffusion Map Embedding (DME—green), Manifold Embedding (ME—red), and Geometric Machine Learning (GML—gold) listed 7758, 14 343, 375 550, and 48 562 diffraction patterns as single particles, respectively. 4564 diffraction patterns are common to the classification obtained by PCA, DME, and ME and colored in gray (a). Panels (d)–(g) show histograms of the predictions $\tilde{f}$ for the diffraction patterns indexed with (d) ME, (e) DME, (f) PCA, and (g) the common list. Venn diagrams of GML predictions are shown in (b) and (c). For the GML histogram in (h), we only considered $\tilde{f}\;\in [0.8,\;1.2]$, and those bounds are marked with dotted black lines.

To compare our GML approach with other single-particle identification algorithms, we retrieved the $\tilde{f}$ values for three subsets of diffraction patterns that have previously been classified as single particles that are publicly available.^43^ These subsets were tagged as single particles by three different research groups [Fig. 7(a)] and correspond to 37 550, 14 343, and 7758 diffraction patterns identified by Manifold Embedding (ME),^26^ Diffusion Map Embedding (DME),^16^ and Principal Component Analysis (PCA),^28^ respectively. We also compiled a fourth subset, which contained the single-particle selection common to all three methods. The histograms presented in Figs. 7(d)–7(g) show that the GML predictions mostly agree with all four subsets, i.e., for each subset, the majority of selected snapshots are located near the region of single particles ($\tilde{f}\approx 1$). For comparison with GML, we provide Venn diagrams [Figs. 7(b) and 7(c)] that show the common predictions of GML using the other methods. For the GML prediction [Fig. 7(h)], we considered the entire right peak of the histogram presented in Fig. 6(a).

**FIG. 8.**
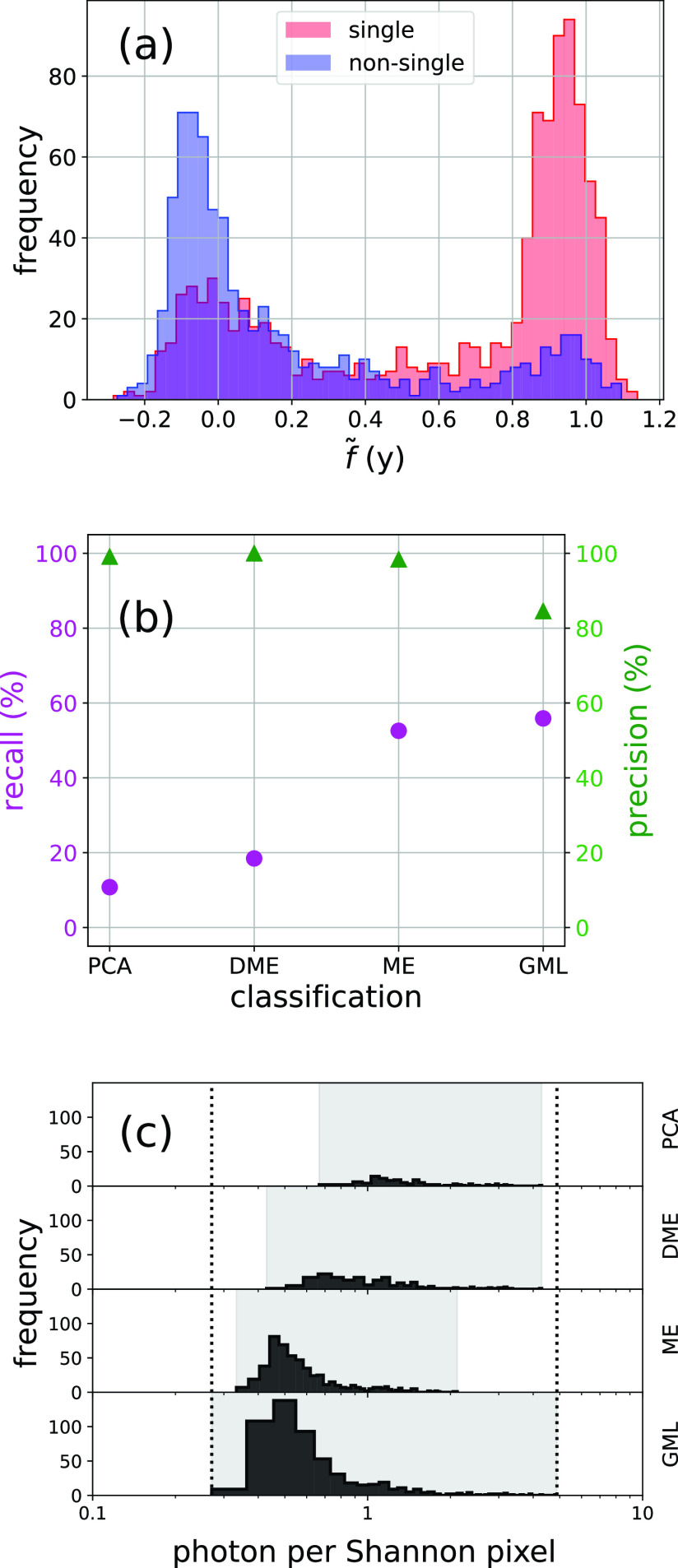
Manual classification of the experimental subset. Panel (a) overlays histograms of prediction values $\tilde{f}$ for a subset of 1779 randomly selected diffraction patterns. The red transparent histogram refers to 1029 ground truth single particles, the blue transparent histogram refers to 750 ground truth non-single particles, and purple color is an overlap between both transparent histograms. Panel (b) plots the prediction quality from different single-particle identification methods in terms of recall (purple) and precision (green). Panel (c) shows histograms of photon counts for ground truth single particles identified by different methods. Light gray backgrounds mark the minimum and maximum photon count ranges for each method. Dotted lines mark the minimum and maximum photon count range for the 1029 ground truth single-particle subset. GML, ME, DME, and PCA refer to Geometric Machine Learning, Manifold Embedding, Diffusion Map Embedding, and Principal Component Analysis, respectively.

To provide a fair ground truth comparison among the different methods, we used a small subset of 1779 diffraction patterns that were randomly chosen and manually classified by an expert into the categories: singles (1029 diffraction patterns) and non-singles (750 diffraction patterns). First, we observe an overall agreement between the predictions $\tilde{f}$ and the manual ground truth classification [Fig. 8(a)]: ground truth single particles are mostly located near $\tilde{f}\approx 1$ and ground truth non-single particles are clustered around $\tilde{f}\approx 0$. Nevertheless, we identified two minor distribution peaks of miscategorized diffraction patterns: a group of ground truth single particles at $\tilde{f}\approx 0$ and another group of ground truth non-single particles near $\tilde{f}\approx 1$.

Next, we quantify the prediction quality of all these algorithms in terms of recall and precision. For the GML method, we considered the subset of diffraction patterns with $\tilde{f}$ values from 0.8 to 1.2. Figure 8(b) shows that the recall (purple dots) by GML is 55.88%, outperforming the ME, DME, and PCA methods that have recalls of 52.58%, 18.46%, and 10.79%, respectively. However, the precision is lowest for GML [Fig. 8(b)—green triangles] with a value of 84.55%, whereas ME, DME, and PCA yield subsets with very high precisions of 98.36%, 100%, and 99.11%. The low precision for GML is due to the larger number of false positives: 105 ground truth non-single particles are mislabeled as single particles ($\tilde{f}\;\in [0.8,\;1.2]$), whereas ME, DME, and PCA mislabel only 9, 0, and 1 ground truth non-single particles. We attribute this contamination caveat to the simplified representation in the training dataset, which does not fully capture all the complexity of the non-single experimental diffraction patterns [compare Figs. 1(e)–1(h) with Figs. 6(d) and 6(e)]. This apparent disadvantage of the GML approach could be reduced either by considering a prediction range narrower than $\tilde{f}\;\in [0.8,\;1.2]$ at the cost of the recall or by including a more realistic representation of non-singles.

After that, we compare the photon count ranges that can be predicted by each method [Fig. 8(c)]. It is worth mentioning that the ME and PCA methods applied thresholding before the classification to exclude outliers with high and low photon counts, respectively. The photon count limits for the 1029 ground truth single particles are pictured in dotted lines. The GML method identifies 575 ground truth single particles that cover the entire photon count range, including the low photon count region where single-particle identification is a challenge. The other single-particle identification methods select different numbers of ground truth single particles: 541 for ME, 190 for DME, and 110 for PCA; however, these methods only cover limited regions of the photon count [Fig. 8(c)—gray backgrounds].

**FIG. 9.**
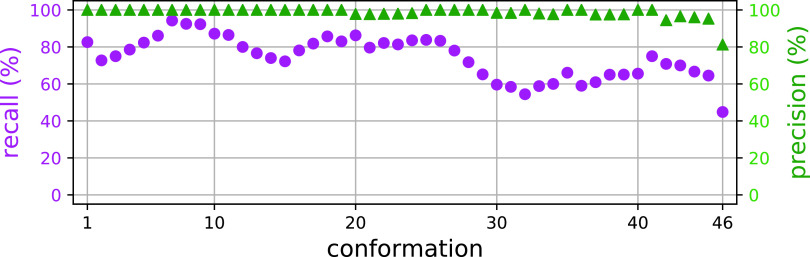
Conformational variability. Plots show the recall (purple circles) and precision (green triangles) for GML predictions using experimental PR772 diffraction patterns that correspond to different molecular conformations.

As a final analysis, we examined the prediction $\tilde{f}$ for diffraction patterns of single particles with varying structural conformations. In a previous study, Hosseinizadeh *et al.*^6^ reported multiple PR772 structures from the same experimental dataset. These structures were sorted into a classification that shows the growth of a tubular structure from an icosahedral vertex. Such a conformational change is needed for the reorganization and release of the viral genome. We retrieved the $\tilde{f}$ values in our small subset of 1779 diffraction patterns that also belong to 46 subset classifications that show moderate conformational changes.^6^ Figure 9 shows the results. The conformations are numbered according to their deviation from the icosahedral shape (conformation 1) to an asymmetric capsid with a tubular protrusion (conformation 46). Both precision and recall show a trend downward, with the highest values near the icosahedral shape (conformation 1). For the conformation with the largest protrusion (conformation 46), the precision and recall have their lowest values. It is worth highlighting that the precision values are all above 80% and the recall values range from 40% to above 90%. Thus, we consider GML a tolerant method, which allows identification of single particles with different conformations. In the supplementary material—Sec. S6, we include the distribution of the ground truth and $\tilde{f}$ values for all conformations.

## CONCLUSIONS

In this work, we presented an efficient single-particle identification method based on Geometric Machine Learning. Our approach employs a noise-free training dataset of XFEL diffraction patterns of a mixture of single and multiple particles to create a template diffusion map for Machine Learning. Then, test datasets under various conditions that are common in experimental beamtimes (varying signal-to-noise levels, background signals, and intensity variations due to shot-to-shot impact conditions) are classified into bimodal distributions of singles and non-singles.

GML retrieves a large number of single-particle diffraction patterns, and its performance is better than previously used methods in terms of recall [Fig. 8(b)]. This is of central importance for obtaining high-resolution structures of biological entities, as the resolution of the reconstruction depends critically on the number of single-particle diffraction patterns, which is needed to overcome the low signal levels.^25^ In particular, GML outperforms existing methods in the low photon count regime, where single-particle identification is most difficult. Another advantage of GML is its robustness in the presence of structural variability; sorting single-particle conformations can subsequently be used to compile molecular movies and calculate energy landscapes.

As expected, the GML approach is not free of caveats and there is room for improvement. First, as the diffraction patterns in the training dataset stem from a known static structure, the question then arises as to why identify single-particle snapshots for a structure already known. However, the static structure is an average over many conformations and GML enables us to deal with structural heterogeneity. In the case of a de novo biomolecule, a training dataset can still be prepared based on an approximate model at low resolution. Second, GML retrieves a high number of false positives, which limits the predictions. As mentioned before, we attribute this partially to our oversimplified representation of multiple particles, and the number of false positives can be decreased with either a better representation of non-singles or a narrower threshold selection. Third, we used only one annular region for identification of single particles, whose bounds ($q\in \left[0.029,0.081\right]$) were chosen to emphasize the high frequency features present in diffraction patterns of single particles and to avoid their angular isotropy at low q. However, multiple particles also have their own characteristic features at low q, e.g., modulated patterns due to interparticle interference, which can also be included. Overall, these three limitations can be addressed by refining the structural information in the training dataset. Although our supervised single-particle identification method requires some *a priori* structural information, single particles for a de novo structure can still be determined by either using low resolution structures or refining the multiple particle representations in the training dataset.

A critical aim for SPI with XFELs is to obtain structural information from biomolecules at high resolution. Ongoing advances in sample delivery and reduction of background scattering are aimed to increase the yield and signal-to-noise ratio of the diffraction patterns.^46^ These improvements are implemented together with the new generation of high-repetition rate XFELs^47,48^ which can generate datasets of tens of millions of diffraction patterns during a single experimental beamtime. Our GML approach allows for efficient analysis of such large datasets and recalls significantly large numbers of single-particle diffraction patterns, as required for high-resolution reconstruction. Moreover, compared to other identification methods, a major advantage of GML is its ability to retrieve single particles in the presence of structural variability. Sorting different molecular conformations will allow researchers to assemble three-dimensional molecular movies in conjunction with energy landscapes. Altogether, this enables us to obtain structural information well beyond static structures and can uncover the function of biological molecules.

## SUPPLEMENTARY MATERIAL

See the supplementary material for additional details about (1) generation of simulated diffraction patterns, (2) number of orientations chosen to give a representative distribution of single-particle diffraction patterns, (3) pre-processing of diffraction patterns, (4) movie of prediction as a function of the number of eigenvectors, (5) ROC and purity plots for the simulated test dataset, (6) distribution of the ground truth and $\tilde{f}$ values for different molecular conformations, and (7) computational performance.

## Data Availability

The data that support the findings of this study are available from the corresponding author upon reasonable request. The experimental XFEL single-particle data of the virus PR772 were collected at LCLS and are openly available from the Coherent X-ray Imaging Data Bank (CXIDB) at https://doi.org/10.11577/1349664, Ref. 16.
